# Validity and Reliability of a Phone App and Stopwatch for the Measurement of 505 Change of Direction Performance: A Test-Retest Study Design

**DOI:** 10.3389/fphys.2021.743800

**Published:** 2021-09-24

**Authors:** Zhili Chen, Chao Bian, Kaifang Liao, Chris Bishop, Yongming Li

**Affiliations:** ^1^School of Physical Education & Sport Training, Shanghai University of Sport, Shanghai, China; ^2^Faculty of Science and Technology, London Sport Institute, Middlesex University, London, United Kingdom; ^3^China Institute of Sport Science, Beijing, China

**Keywords:** adolescent, basketball, change of direction, smartphone, timing device

## Abstract

**Purpose:** The aim of this study was to explore the validity and reliability of a phone app [named: change of direction (COD) timer] and stopwatches for the measurement of COD performance.

**Methods:** Sixty-two youth basketball players (age: 15.9±1.4yrs., height: 178.8±11.0cm, and body mass: 70.0±14.1kg) performed six trials of 505 COD test (with the left side being the plant leg first, then the right side). The completion time was measured simultaneously *via* timing gates (with error correction processing algorithms), the phone app, and stopwatches.

**Results:** There was an almost perfect correlation and agreement between timing gates and COD timer (*r*=0.978; SEE=0.035s; and LoA=−0.08~0.06s), but a lower correlation and agreement between timing gates and stopwatch (*r*=0.954; SEE=0.050s; and LoA=−0.17~0.04s) with statistical significance in completion time (ES=1.29, 95%CI: 1.15–1.43, *p*<0.01). The coefficient of variation revealed similar level of dispersion between the three timing devices (timing gates: 6.58%; COD timer: 6.32%; and stopwatch: 6.71%). Inter-observer reliability (ICC=0.991) and test-retest reliability (ICC=0.998) were excellent in COD timer, while the inter-observer reliability was lower (ICC=0.890) in the stopwatches.

**Conclusion:** In the 505 COD test, the COD timer was able to provide a valid and reliable measurement. On the contrary, stopwatch was not recommended because of large error. Thus, if timing gates are unavailable, practitioners can adopt the COD timer app to assess 505 COD speed times.

## Introduction

Change of direction (COD)encompasses the skills and abilities needed to change movement direction, velocity, or modes (Nimphius et al., 2017), which plays a pivotal role in match-winning situations of team sports (Taylor et al., 2017; Wen et al., 2018; Loturco et al., 2019; Stojanović et al., 2019). Naturally, evaluating players’ COD performance has received great attention from coaches and sports scientists (Baker and Newton, 2008; Chaouachi et al., 2012; Nimphius et al., 2017). The 505 COD test is one of the developed protocols to measure COD performance and involves a high-intensity cut which is often performed in competitions, thus is widely applicable to many team or racquet sports (Gabbett et al., 2008; Stewart et al., 2014; Nimphius et al., 2016). In addition, by measuring the completion time of left and right sides (as defined by the plant limb), the 505 COD test can be used to assess the imbalance between limbs (Wen et al., 2018). Furthermore, the duration of the 505 COD test is relatively short (2–3s; Draper and Lancaster, 1985; Sayers, 2015) in comparison with other tests (~13s; Pauole et al., 2000; Lockie et al., 2013, 2014; Wilkinson et al., 2019), which means it places more emphases on COD ability (i.e., some COD tests have come under criticism for having sections of the test overly focused on linear sprinting ability; Nimphius et al., 2016).

In practice, electronic timing gates, radar gun, and photo-finish camera technology have been extensively adopted as the gold standard instruments for timing the 505 COD test (Haugen and Buchheit, 2016; Altmann et al., 2018). However, the high cost associated with these methods can make it challenging for practitioners with limited budgets. Meanwhile, the stopwatch is a more portable and less expensive alternative with acceptable relative reliability (ICC=0.92–0.99; Hetzler et al., 2008; Mayhew et al., 2010). Although previous studies have indicated that manual timing has large absolute errors during linear sprinting tasks (Brechue et al., 2008; Haugen et al., 2016), no study has explored the validity and reliability of stopwatch in COD tests.

More recently, some cost-effective smartphone apps have been developed to measure various components of physical performance, such as vertical jump height or barbell velocity based on the slow-motion function of cameras, and proved to be practical and accurate (Gallardo-Fuentes et al., 2016; Balsalobre-Fernández et al., 2018; Haynes et al., 2019; Perez-Castilla et al., 2021). Among these, the COD timer app was specially developed to measure the completion time during the COD test and has been supported to be valid and reliable by the developers (Balsalobre-Fernández et al., 2019). However, the study only investigated the COD timer app in 5+5 COD test, and it is questionable whether its findings can be applied to other COD tests due to the different starting styles between tests (i.e., static vs. flying). Furthermore, the validity and reliability of the COD timer app has not been investigated by a third party other than the developers themselves. Furthermore, several meaningful measures of reliability (e.g., inter-observer and test-retest reliability), which may impact recorded completion time, have not been reported. Therefore, the aim of the present study was to assess the validity and reliability of the COD timer and the stopwatch using the 505 COD test. We hypothesized that the COD timer app would be a valid and reliable alternative for the measurement of completion time in the 505 COD test, with better validity and reliability than the stopwatch.

## Materials and Methods

### Participants

Sixty-two healthy, youth basketball players (age 15.9±1.4yrs., height 178.8±11.0cm, and body mass 70.0±14.1kg) with at least 4years of basketball training experience volunteered to participate in this study. Based on the work of Balsalobre-Fernández et al. (2019), a minimum sample size of 42 was determined from an *a priori* power analysis using G*Power (Version 3.1, University of Dusseldorf, Germany) based upon an effect size of 0.19, a power of 0.95, alpha level of 0.05, and correlation among repeated measures of 0.964. Prior to the study, the subjects were informed of the test procedure and the potential risk. Written informed consent was obtained from participants and their coaches in advance. Ethics approval was provided by the Shanghai University of Sport.

### Design and Procedures

The present study used an observational design where data were completed for the 505 COD test in a single session. All the trials were timed simultaneously *via* the timing gates (Smartspeed pro, Fusion sport, Australia), a phone app (COD timer, Apple Inc., United States), and three different individuals using stopwatches (SW141, Sekio, Japan), and the results were compared in order to perform validity and reliability analysis with statistical procedures. Times were measured to the nearest 0.01s. All tests were performed during the afternoon (4p.m.~6p.m.) in similar temperature (24~26°C) and humidity (76~80%) conditions in 2days.

### Instruments

#### Timing Gates

A pair of timing gates with error correction processing algorithms (Smartspeed pro, Fusion sport, Australia) were placed at the finish line. A distance of 2m was adjusted between the infrared transmitter and the reflector. The height was set at approximately 0.9m off the ground, corresponding to subjects’ hip height as previously recommended and to avoid the timing gates being triggered prematurely by a swinging arm or leg (Dos’Santos et al., 2019). The timing gates with error correction processing (ECP) algorithms sampling at 1000Hz (accuracy to 1/1000th of a second) was considered as reference to measure the completion time of the trials in this study (Strutzenberger et al., 2016; Altman et al., 2018).

#### COD Timer

The COD timer app was installed on an iPAD (iPAD pro, Apple Inc., United States) with IOS 14.0 operative system. The iPAD was placed in a tripod 6m away, perpendicular from the lane, and in line with the finishing line. The iPAD recorded the video of all trials. The start and finish of each trial was considered as the first frame in which the subject crossed the timing gates with their torso. Two authors from this study analyzed all the video independently twice, 1week apart.

#### Stopwatch

Three experienced timers stood perpendicular to, and 3m away from the lane, with their position in line with the finish line. They were instructed to start and stop their watches independently when the subject’s torso passed through the finish line based on their visual perception (Mann et al., 2015). No communication was allowed among timers during the test.

### 505 COD Test

**Figure 1:**
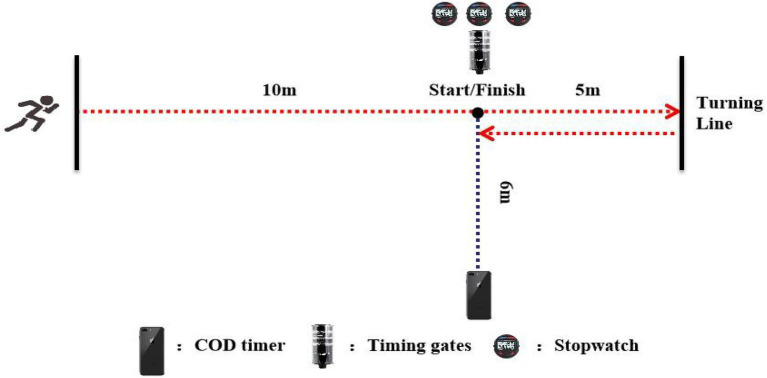
Layout of the 505 COD test.

Every subject performed 505 COD test with a total of six trials (three trials with the left side being the plant leg first, then the right side for the remaining trials). The test was initiated by each subject and a 3-min rest was provided between each trial. All tests were conducted on a wooden basketball court to ensure ecological validity to the subjects’ playing environment. Familiarization was conducted 1week prior to the formal test, where players were allowed to practice the 505 test, under the supervision of the primary researcher. Prior to the trials, subjects completed a standardized 15-min warm-up protocol including jogging, dynamic stretching (two sets of four knee hug-moving, four walking quad stretches, two inchworms, and two world’s greatest stretch on each side), and activation exercises (2×maximal effort runs for 5s). After that, subjects performed the 505 COD test. During the 505 COD test, the subjects started from the start line with a standing posture, sprinted through the vertical marker, reached the turning line, turned 180°, and re-accelerated to pass the finishing line as fast as possible (Figure 1).

### Statistical Analyses

IBM SPSS Statistics 26 for windows (IBM Co., United States) and JASP 0.9.2 for windows (University of Amsterdam, Netherlands) were used to analyze the data. Validity analysis included two observers (phone app) and three timers (stopwatch) compared to the electronic timing gates in six trials of the 505 COD test. A linear regression with Pearson’s *r* correlation coefficient, the standard error of the estimate (SEE), and the slope of the regression line was analyzed to assess the concurrent validity of the COD timer app and stopwatch, in comparison with the timing gates. Also, to test collinearity, the Durbin-Watson test was used. The strength of the *r* coefficients was interpreted as follows: trivial (<0.10), small (0.10–0.29), moderate (0.30–0.49), high (0.50–0.69), very high (0.70–0.89), or practically perfect (>0.90; Hopkins et al., 2009). Paired samples *t*-tests and Bland-Altman plots were used to identify potential systematic bias *via* mean bias and the regression line on the Bland-Altman plots (Bland and Altman, 1986). Cohen’s *d* was used to assess the mean differences between the measures obtained with each instrument, which was rated as trivial (<0.2), small (0.2–0.59), moderate (0.6–1.19), or large (1.2–2.0; Rhea, 2004). Paired samples *t*-tests and Cohen’s *d* effect sizes (with 95% confidence intervals) were also calculated to identify mean differences between observers. A one-way ANOVA with Bonferroni post-hoc testing was used to evaluate the differences between the three hand timers. The coefficient of variation (CV) was used to analyze the stability of timing systems, with a CV<10% considered as acceptable reliability (Atkinson and Nevill, 1998). The interclass correlation coefficient with 95%CI (ICC, two-way random, and absolute agreement) was used to assess the test-retest reliability (phone app) and inter-observers’ reliability (phone app and stopwatch). ICC was interpreted as following: poor (<0.50), moderate (0.50–0.74), good (0.75–0.89), and excellent (>0.9; Koo and Li, 2016). The level of significance was set at *p*≤0.05.

## Results

After excluding invalid data, such as slip (three cases) or blurred images (caused by the failure to focus the phone’s lens in time; two cases), a total of 367 trials and 1,101 cases were included in the final analysis. All mean date are presented in Table 1.

**Table 1:** The average time obtained by timing devices, session, observers, and timers, from trial one to trial six in 505 COD test.

Devices	Left			Right			Total
	Trial 1	Trial 2	Trial 3	Trial 4	Trial 5	Trial 6	
Timing gate:	2.54 ± 0.17	2.51 ± 0.16	2.52 ± 0.16	2.53 ± 0.16	2.53 ± 0.17	2.52 ± 0.15	2.52 ± 0.16
phone app:	2.54 ± 0.16	2.53 ± 0.17^b^	2.52 ± 0.18^b^	2.54 ± 0.17	2.55 ± 0.16^b^	2.53 ± 0.16^b^	2.53 ± 0.16^b^
*Observer1 (1st)*	2.54 ± 0.16	2.53 ± 0.17^b^	2.52 ± 0.18^b^	2.54 ± 0.17	2.54 ± 0.16^b^	2.53 ± 0.16^b^	2.53 ± 0.16^b^
*Observer1 (2nd)*	2.54 ± 0.16	2.53 ± 0.17^b^	2.52 ± 0.18^b^	2.54 ± 0.17	2.55 ± 0.16^b^	2.53 ± 0.16^b^	2.53 ± 0.16^b^
*Observer2 (1st)*	2.54 ± 0.16	2.53 ± 0.17^b^	2.52 ± 0.18^b^	2.54 ± 0.16	2.55 ± 0.16^b^	2.53 ± 0.15^b^	2.53 ± 0.16^b^
*Observer2 (2nd)*	2.54 ± 0.16	2.52 ± 0.17^b^	2.52 ± 0.18^b^	2.54 ± 0.16	2.54 ± 0.16^b^	2.53 ± 0.15^b^	2.53 ± 0.16^b^
Stopwatch:	2.56 ± 0.19^a^	2.55 ± 0.18^a^	2.56 ± 0.19^b^	2.56 ± 0.17^a^	2.58 ± 0.17^b^	2.56 ± 0.16^b^	2.56 ± 0.18^b^
*Timer1*	2.61 ± 0.19^b^	2.59 ± 0.18^b^	2.60 ± 0.20^b^	2.62 ± 0.18^b^	2.60 ± 0.18^b^	2.59 ± 0.16^b^	2.60 ± 0.18^b^
*Timer2*	2.54 ± 0.17^a^	2.51 ± 0.16^b^	2.52 ± 0.16^b^	2.53 ± 0.16^a^	2.53 ± 0.17^b^	2.52 ± 0.15^b^	2.52 ± 0.16^b^
*Timer3*	2.54 ± 0.16^b^	2.53 ± 0.17^b^	2.52 ± 0.18^b^	2.54 ± 0.17^b^	2.55 ± 0.16^b^	2.53 ± 0.16^b^	2.53 ± 0.16^b^

*First, the data obtained from the first manipulate session; second, the data obtained from the second manipulate session*.

a *Significantly (P<0.05) different from the timing gate.*

b *Significantly (P<0.01) different from the timing gate*.

### Concurrent Validity

#### COD Timer

**Figure 2:**
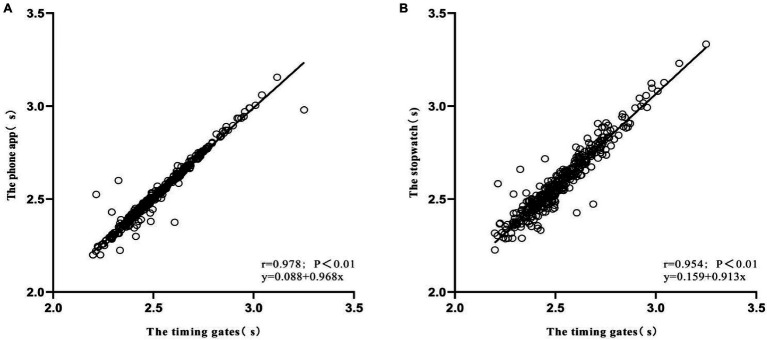
Linear relationship between devices for the completion time: **(A)** phone app and timing gates; **(B)** stopwatch and timing gates.

**Figure 3:**
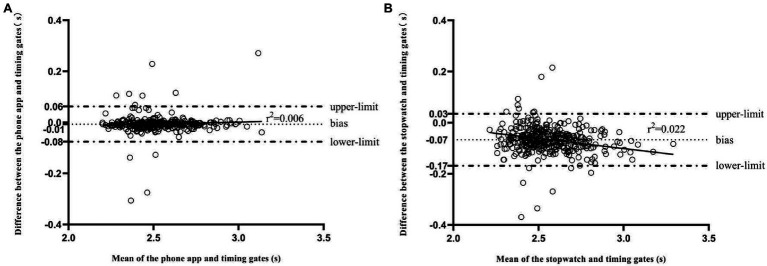
Bland-Altman plots for the measurement of completion time between devices: **(A)** phone app and timing gates; **(B)** stopwatch and timing gates. The central thin dashed line represents the absolute average difference between devices, and upper and lower horizontal lines represent the observed bias with 1.96 standard deviations (SD), while the solid line is the regression line of the residual.

The COD timer exhibited excellent concurrent validity in 505 COD test in comparison with timing gates (*r*=0.978; SEE=0.035s; and slope of the regression line=0.968; *p*<0.001; Figure 2). No collinearity was observed in the Durbin-Watson test (d=2.4). Significant but trivial difference was observed between the COD timer and timing gates (Mean difference=0.007s; *d*=0.19, 95% CI=0.09–0.29; *p*<0.001). The mean bias and 95% limits of agreement (−0.01s, 95% CI=−0.08s-0.06s) between the COD timer and timing gates revealed a trivial difference. The regression line in the Bland-Altman plot showed no heteroscedasticity in the distribution of the difference between devices as revealed by its regression line (*r^2^*=0.006; Figure 3).

#### Stopwatch

Pearson’s correlation coefficient showed a very high relationship between the completion time measured with stopwatch and the timing gates (*r*=0.954, SEE=0.05s, and slope of the regression line=0.913, *p*<0.001). No collinearity was observed as revealed by the Durbin-Watson test (*d*=2.2). Significant and large differences were observed between the stopwatch and the timing gates (Mean difference=0.067s; *d*=1.29, 95% CI=1.15–1.43; *p*<0.001). A systematic bias between the stopwatch and the timing gates (Bias=0.07s; 95% LoA=−0.07–0.13s) was found by the analysis of the Bland-Altman plot. Finally, the regression line in the Bland-Altman plot showed significant heteroscedasticity in the distribution of the difference between devices (*r*^2^=0.022; Figure 3). There were significant and moderate to large differences between timers and timing gates (timer1: *d*=1.18, 95% CI=1.05–1.31; timer2: *d*=0.61, 95% CI=0.50–0.72; and timer3: *d*=1.12, 95% CI=0.99–1.25).

### Reliability

Three timing devices all showed acceptable reliability in 505 COD test (CV: timing gates=6.35%; COD timer=6.32%; and stopwatch=6.95%). The COD timer showed non-significant, trivial, and near perfect agreement in inter-observers’ reliability (*p*=0.419, *d*=0.04, 95% CI=−0.06–0.15; ICC=0.991, 95% CI=0.990–0.992) and test-retest reliability (*p*=0.06, *d*=0.10, 95% CI=0.03–0.17; ICC=0.998, 95% CI=0.998–0.999) for the measurement of the 505 COD test. For the stopwatch condition, although ICC values were good (ICC=0.890, 95% CI=0.838–0.922), timers reported significant differences between each other (*p*<0.001), with the second timer being faster than the other two (*p*<0.001).

## Discussion

The aim of this study was to assess the concurrent validity, inter-observer agreement, and test-retest reliability of the COD timer smartphone app and stopwatch in measuring 505 COD completion time. Results showed that the COD timer was highly valid and reliable and can be an appropriate alternative for more economical and portable measurement of COD performance. In contrast, the stopwatch should be avoided due to large measurement errors in 505 COD test.

Compared to the traditional laboratory equipment, smartphone apps have the advantage of being easily affordable to all practitioners as well as being easy to operate, which makes them increasingly viable for sports researchers and fitness coaches (Peart et al., 2019). In agreement with Balsalobre et al. (*r*=0.964; 95%CI=0.95–1.00) and Romero-Franco et al. (*r*=0.989–0.999), who observed a high agreement with no significant differences compared to timing gates for the measurement of completion time when using a smartphone app in 5+5 test and 40m sprint (Romero-Franco et al., 2017; Balsalobre-Fernández et al., 2019), our results also revealed a very high concurrent validity of the COD timer app with respect to the timing gates. The linear regression analysis showed a very high association (*r*=0.978) and the slope coefficient was very close to the identity line (slope=0.968). Simply put, this means that the values measured with both devices were highly consistent and this was supported by the data presented in Bland-Altman plots. Most of the values were close to the mean of the differences between instruments, and the analysis of the regression line between the data points showed a very low *r^2^* value of 0.006, with a slope close to 0, indicating that the differences between devices were almost negligible. However, significant differences in completion time were observed between COD timer app and the timing gates (*p*<0.01), which could be explained by the fact that the sample size was calculated specific to the power analysis, and might be inflated by the type error I to make false inference due to the large number of records. However, when interpreting the effect size data, only trivial differences were evident. Regardless, all of the aforementioned conclusions have been made possible by rapid advancements in technology and have greatly enhanced the functions of smartphones. This is evidenced by the ability to record videos at 240 frames per second (fps) and 1080p quality. In fact, the potential problem of the COD timer app is that the observer needs to select the start and finishing frames manually, which in turn may cause measurement error when calculating completion time. That said, our results suggest that with frame-by-frame analysis, the manual measurement error does not influence the concurrent validity.

Meanwhile, a high level of reliability is also necessary for timing devices. To our knowledge, only few studies have considered the inter-observer reliability of the smartphone app. Romero-Franco et al., when testing 40m sprint, showed a near perfect agreement (ICC=0.998, 95% CI=0.997–0.998) and no significant differences between two independent observers (mean difference=0.004±0.03, *p*=0.999). Balsalobre-Fernández et al. also saw a high level of inter-observer agreement when measuring the mean velocity of barbell (ICC=0.941, 90% CI=0.922–0.955; Balsalobre-Fernández et al., 2018) and the height of CMJ (ICC=0.999, 95% CI=0.998–0.999; Balsalobre-Fernández et al., 2015). Similarly, we found that the level of agreement between the two observers was also very high (ICC=0.991), and inter-observers’ differences were not significant with trivial effect size (mean difference=0.007s, *p*=0.419, *d*=0.04). The similar findings seen in these studies and the current study suggest the reliability of the slow-motion apps has been confirmed, and it highlights the usability of the COD timer app. Furthermore, after 1week, we repeated the operation process of COD timer app to analyze the saved videos again. The result revealed near perfect consistency between the first and the second operation sessions in completion time (ICC=0.998; Table 1). From a practical standpoint, this means that the practitioners can assess the video repeatedly using the COD timer and it is plausible for them to analyze a large number of trials, when convenient for them. Continuing on this practical theme, it should be noted that the COD timer app only costs 11 USD, which is far cheaper than timing gates and equal to the cost of a stopwatch. Taken together, the COD timer can be considered as a valid, reliable, and cost-effective alternative for practitioners who need to measure the 505 COD test, but without availability of the more expensive electronic timing gates.

On the contrary, although the stopwatch showed high correlation with timing gates (*r*=0.954), a large difference was found in completion time (mean difference=0.067s, *p*<0.01, *d*=1.29). This was further supported by the poor agreement (*r*^2^=0.022) *via* the regression line in Bland-Altman plot. Interestingly, our study demonstrated that the stopwatch was always slower than timing gates in 505 COD test with a small difference (~0.07s). This was actually in contrast to the previous literature which has reported faster times in stopwatches, with differences approximately 0.20~0.24s compared to electronic timing systems (Brechue et al., 2008; Hetzler et al., 2008; Mayhew et al., 2010; Mann et al., 2015). To the authors’ knowledge, no previous studies have explored the validity and reliability of a stopwatch during a COD test. Although the correlation between the stopwatch and timing gates was classified as excellent, the large absolute error still cannot be considered acceptable. Actually, the discrepancy of elite and average players in speed performance is relatively small, the time difference between stopwatch and timing gates is close to the 50th and 10th percentile over 10m sprint in male soccer players (Haugen et al., 2014). The relevance here being that if practitioner opts to use a stopwatch, the large differences in reaction times may actually mask the inherent variations in COD performance often seen within a group of athletes.

Despite the novelty and usefulness of the present study, there were a few limitations which should be acknowledged. First, the conclusion of this study can only be applied to the 505 COD test, future research should determine the validity and reliability across other tests, such as the pro-agility test. Second, the COD timer can only be applied to the IOS operation system. Thus, it is necessary to develop an equivalent for android smartphones, which would increase the usability of the app in the field.

## Applications and Conclusion

The accuracy and repeatability are essential for timing systems when measuring in 505 COD test. The results of the present investigation add to the literature that such short completion time in 505 COD test can be easily, validly, and reliably measured using slow-motion video analysis by COD timer which is available on the App store (Apple Inc., United States). By contrast, stopwatch is not recommended because of the large measurement errors between timing gates and each timers.

## Data Availability Statement

The original contributions presented in the study are included in the article/supplementary material, and further inquiries can be directed to the corresponding author.

## Ethics Statement

Prior to the study, the subjects were informed of the test procedure and the potential risk. Written informed consent was obtained from each participant and their parents/coaches in advance. Ethics approval was provided by the Shanghai University of Sport.

## Author Contributions

CZ: acquisition of data, conception and design of study, analysis of data, and drafting the manuscript. BC: acquisition of data. LK, CB, and LY: revising the manuscript. All authors contributed to the article and approved the submitted version.

## Funding

This study was supported by the Ministry of Science and Technology of the People’s Republic of China (2018YF0300901) and the Shanghai Science and Technology Commission (TP2017063).

## Conflict of Interest

The original contributions presented in the study are included in the article/supplementary material, further inquiries can be directed to the corresponding author.

## Publisher’s Note

All claims expressed in this article are solely those of the authors and do not necessarily represent those of their affiliated organizations, or those of the publisher, the editors and the reviewers. Any product that may be evaluated in this article, or claim that may be made by its manufacturer, is not guaranteed or endorsed by the publisher.

## References

[ref1] Altmann S. Ringhof S. Becker B. Woll A. Neumann R. (2018). Error-correction processing in timing lights for measuring sprint performance: does it work? Int. J. Sports Physiol. Perform. 26, 1–3. doi: 10.1123/ijspp.2017-0596 29809060

[ref2] Atkinson G. Nevill A. M. (1998). Statistical methods for assessing measurement error (reliability) in variables relevant to sports medicine. Sports Med. 26, 217–238. doi: 10.2165/00007256-199826040-00002, PMID: 9820922

[ref3] Baker D. G. Newton R. U. (2008). Comparison of lower body strength, power, acceleration, speed, agility, and sprint momentum to describe and compare playing rank among professional rugby league players. J. Strength Cond. Res. 22, 153–158. doi: 10.1519/JSC.0b013e31815f9519, PMID: 18296969

[ref4] Balsalobre-Fernández C. Bishop C. Beltrán-Garrido J. V. Cecilia-Gallego P. Cuenca-Amigó A. Romero-Rodríguez D.. (2019). The validity and reliability of a novel app for the measurement of change of direction performance. J. Sports Sci. 37, 2420–2424. doi: 10.1080/02640414.2019.1640029, PMID: 31272332

[ref5] Balsalobre-Fernández C. Glaister M. Lockey R. A. (2015). The validity and reliability of an phone app for measuring vertical jump performance. J. Sports Sci. 33, 1574–1579. doi: 10.1080/02640414.2014.996184, PMID: 25555023

[ref6] Balsalobre-Fernández C. Marchante D. Muñoz-López M. Jiménez S. L. (2018). Validity and reliability of a novel phone app for the measurement of barbell velocity and 1RM on the bench-press exercise. J. Sports Sci. 36, 64–70. doi: 10.1080/02640414.2017.1280610 28097928

[ref7] Bland J. M. Altman D. G. (1986). Statistical methods for assessing agreement between two methods of clinical measurement. Lancet 1, 307–310. PMID: . doi: 10.1016/S0140-6736(86)90837-8 2868172

[ref8] Brechue W. F. Mayhew J. L. Piper F. C. Houser J. J. (2008). Comparison between hand- and electronic-timing of sprint performance in college football players. Mo J Hlth Phys Educ Rec Dance. 18, 50–58.

[ref9] Chaouachi A. Manzi V. Chaalali A. Wong del P. Chamari K. Castagna C. (2012). Determinants analysis of change-of-direction ability in elite soccer players. J. Strength Cond. Res. 26, 2667–2676. doi: 10.1519/JSC.0b013e318242f97a, PMID: 22124358

[ref10] Dos’Santos T. Thomas C. Jones P. A. Comfort P. (2019). Assessing asymmetries in change of direction speed performance: application of change of direction deficit. J. Strength Cond. Res. 33, 2953–2961. doi: 10.1519/JSC.0000000000002438, PMID: 29373434

[ref11] Draper J. Lancaster M. (1985). The 505 test: A test for agility in the horizontal plane. Aust. J. Sci. Med. Sport 17, 15–18.

[ref12] Gabbett T. J. Kelly J. N. Sheppard J. M. (2008). Speed, change of direction speed, and reactive agility of rugby league players. J. Strength Cond. Res. 22, 174–181. doi: 10.1519/JSC.0b013e31815ef700, PMID: 18296972

[ref13] Gallardo-Fuentes F. Gallardo-Fuentes J. Ramírez-Campillo R. Balsalobre-Fernández C. Martínez C. Caniuqueo A.. (2016). Intersession and intrasession reliability and validity of the my jump app for measuring different jump actions in trained male and female athletes. J. Strength Cond. Res. 30, 2049–2056. doi: 10.1519/JSC.0000000000001304, PMID: 27328276

[ref14] Haugen T. Buchheit M. (2016). Sprint running performance monitoring: methodological and practical considerations. Sports Med. 46, 641–656. doi: 10.1007/s40279-015-0446-0, PMID: 26660758

[ref15] Haugen T. Tonnessen E. Hisdal J. Seiler S. (2014). The role and development of sprinting speed in soccer. Int. J. Sports Physiol. Perform. 9, 432–441. doi: 10.1123/ijspp.2013-0121, PMID: 23982902

[ref16] Haynes T. Bishop C. Antrobus M. Brazier J. (2019). The validity and reliability of the my jump 2 app for measuring the reactive strength index and drop jump performance. J. Sports Med. Phys. Fitness 59, 253–258. doi: 10.23736/S0022-4707.18.08195-1, PMID: 29589412

[ref17] Hetzler R. K. Stickley C. D. Lundquist K. M. Kimura I. F. (2008). Reliability and accuracy of handheld stopwatches compared with electronic timing in measuring sprint performance. J. Strength Cond. Res. 22, 1969–1976. doi: 10.1519/JSC.0b013e318185f36c, PMID: 18978613

[ref18] Hopkins W. G. Marshall S. W. Batterham A. M. Hanin J. (2009). Progressive statistics for studies in sports medicine and exercise science. Med. Sci. Sports 41, 3–13. doi: 10.1249/MSS.0b013e31818cb278 19092709

[ref19] Koo T. K. Li M. Y. (2016). A guideline of selecting and reporting intraclass correlation coefficients for reliability research. J. Chiropr. Med. 15, 155–163. doi: 10.1016/j.jcm.2016.02.012, PMID: 27330520 PMC4913118

[ref20] Lockie R. G. Schultz A. B. Callaghan S. J. Jeffriess M. D. (2014). The effects of traditional and enforced stopping speed and agility training on multidirectional speed and athletic function. J. Strength Cond. Res. 28, 1538–1551. doi: 10.1519/JSC.0000000000000309, PMID: 24169474

[ref21] Lockie R. G. Schultz A. B. Callaghan S. J. Jeffriess M. D. Berry S. P. (2013). Reliability and validity of a new test of change-of-direction speed for field-based sports: the change-of-direction and acceleration test (CODAT). J. Sports Sci. Med. 12, 88–96. PMID: . doi: 10.1097/BOT.0b013e318251e66d 24149730 PMC3761765

[ref22] Loturco I. Jeffreys I. Abad C. C. C. Kobal R. Zanetti V. Pereira L. A.. (2019). Change-of-direction, speed and jump performance in soccer players: a comparison across different age-categories. J. Sports Sci. 38, 1–7. doi: 10.1080/02640414.2019.1574276 30724662

[ref23] Mann J. B. Ivey P. J. Brechue W. F. Mayhew L. J. (2015). Validity and reliability of hand and electronic timing for 40-yd sprint in college football players. J. Strength Cond. Res. 29, 1509–1514. doi: 10.1519/JSC.0000000000000941, PMID: 25785707

[ref24] Mayhew J. L. Houser J. J. Briney B. B. Williams T. B. Piper F. C. Brechue W. F. (2010). Comparison between hand and electronic timing of 40-yd dash performance in college football players. J. Strength Cond. Res. 24, 447–451. doi: 10.1519/JSC.0b013e3181c08860, PMID: 20072055

[ref25] Nimphius S. Callaghan S. J. Bezodis N. E. Lockie R. G. (2017). Change of direction and agility tests: challenging our current measures of performance. Strength Conditioning J. 40, 26–38. doi: 10.1519/SSC.0000000000000309

[ref26] Nimphius S. Callaghan S. J. Spiteri T. Lockie R. G. (2016). Change of direction deficit: a more isolated measure of change of direction performance than total 505 time. J. Strength Cond. Res. 30, 3024–3032. doi: 10.1519/JSC.0000000000001421, PMID: 26982972

[ref27] Pauole K. Madole K. Garhammer J. Lacourse M. Rozenek R. (2000). Reliability and validity of the T-test as a measure of agility, leg power, and leg speed in college-aged men and women. J. Strength Cond. Res. 14, 443–450.

[ref28] Peart D. J. Balsalobre-Fernández C. Shaw M. P. (2019). Use of mobile applications to collect data in sport, health, and exercise science: A narrative review. J. Strength Cond. Res. 33, 1167–1177. doi: 10.1519/JSC.0000000000002344, PMID: 29176384

[ref29] Perez-Castilla A. Boullosa D. Garcia-Ramos A. (2021). Reliability and validity of the iLOAD application for monitoring the mean set velocity during the back squat and bench press exercises performed against different loads. J. Strength Cond. Res. 35, S57–S65. doi: 10.1519/JSC.0000000000003739, PMID: 33021586

[ref30] Rhea M. R. (2004). Determining the magnitude of treatment effects in strength training research through the use of the effect size. J. Strength Cond. Res. 18, 918–920. doi: 10.1519/14403.1, PMID: 15574101

[ref31] Romero-Franco N. Jiménez-Reyes P. Castaño-Zambudio A. Capelo-Ramírez F. Rodríguez-Juan J. J. González-Hernández J.. (2017). Sprint performance and mechanical outputs computed with an phone app: comparison with existing reference methods. Eur. J. Sport Sci. 17, 386–392. doi: 10.1080/17461391.2016.1249031 27806673

[ref32] Sayers M. G. (2015). Influence of test distance on change of direction speed test results. J. Strength Cond. Res. 29, 2412–2416. doi: 10.1519/jsc.0000000000001045 26049789

[ref33] Stewart P. F. Turner A. N. Miller S. C. (2014). Reliability, factorial validity, and interrelationships of five commonly used change of direction speed tests. Scand. J. Med. Sci. Sports 24, 500–506. doi: 10.1111/sms.12019, PMID: 23176602

[ref34] Stojanović E. Aksović N. Stojiljković N. Stanković N. Scanlan A. T. Milanović Z. (2019). Reliability, usefulness, and factorial validity of change-of-direction speed tests in adolescent basketball players. J. Strength Cond. Res. 3, 3162–3173. doi: 10.1519/jsc.0000000000002666 29927890

[ref35] Strutzenberger G. Moore J. Griffiths H. (2016). Effects of gluteal kinesio-taping on performance with respect to fatigue in rugby players. Eur. J. Sport Sci. 16, 165–171. doi: 10.1080/17461391.2015.1004372 25647686

[ref36] Taylor J. B. Wright A. A. Dischiavi S. L. Townsend M. A. Marmon A. R. (2017). Activity demands during multi-directional team sports: a systematic review. Sports Med. 47, 2533–2551. doi: 10.1007/s40279-017-0772-5, PMID: 28801751

[ref37] Wen N. Dalbo V. J. Burgos B. Pyne D. B. Scanlan A. T. (2018). Power testing in basketball: current practice and future recommendations. J. Strength Cond. Res. 32, 2677–2691. doi: 10.1519/JSC.0000000000002459, PMID: 29401204

[ref38] Wilkinson M. Leedale-Brown D. Winter E. M. (2019). Validity of a squash-specific test of change-of-direction speed. Int. J. Sports Physiol. Perform. 4, 176–185. doi: 10.1123/ijspp.4.2.176 19567921

